# Interindividual differences in Pavlovian influence on learning are consistent

**DOI:** 10.1098/rsos.230447

**Published:** 2023-09-20

**Authors:** Sepehr Saeedpour, Mostafa Minadari Hossein, Ophelia Deroy, Bahador Bahrami

**Affiliations:** ^1^ Department of Electrical and Computer Engineering, University of Tehran, Tehran, Iran; ^2^ Department of Psychology, University of Toronto, Toronto, Canada; ^3^ Faculty of Philosophy, Ludwig Maximilian University, Munich, Germany; ^4^ Munich Center for Neuroscience, Ludwig Maximilian University, Munich, Germany; ^5^ Faculty of General Psychology and Education, Ludwig Maximilian University, Munich, Germany; ^6^ School of Advanced Study, University of London, London, UK

**Keywords:** learning, decision making, Pavlovian bias

## Abstract

Pavlovian influences impair instrumental learning. It is easier to learn to approach reward-predictive signals and avoid punishment-predictive cues than their contrary. Whether the interindividual variability in this Pavlovian influence is consistent across time has been examined by a number of recent studies and met with mixed results. Here we introduce an open-source, web-based instance of a well-established Go–NoGo paradigm for measuring Pavlovian influence. We closely replicated the previous laboratory-based results. Moreover, the interindividual differences in Pavlovian influence were consistent across a two-week time window at the level of (i) raw measures of learning (i.e. performance accuracy), (ii) linear, descriptive estimates of Pavlovian bias (test–retest reliability: 0.40), and (iii) parameters obtained from reinforcement learning model fitting and model selection (test–retest reliability: 0.25). Nonetheless, the correlations reported here are still lower than the standards (i.e. 0.7) employed in psychometrics and self-reported measures. Our results provide support for trusting Pavlovian bias as a relatively stable individual characteristic and for using its measure in the computational understanding of human mental health.

## 1. Introduction

Some years after starting his studies of dogs' salivation in the presence of food and food-related cues, Pavlov learned that experiments somewhat similar to his had been performed in America, not by physiologists but by psychologists. ‘Thereupon’, he recollects in his lectures ‘I studied in more detail the American publications, and now I must acknowledge that the honour of having made the first steps along this path belongs to E. L. Thorndike’. On the first reading, the connection is surprising, as Thorndike was studying instrumental learning—how reward and punishment shape the learning of actions—rather than the association between existing unconditional reflex and new stimuli as Pavlov was. Despite the initial contrast in their approaches the bridge between their studies became evident in later research.

Estes [1,2] observed that animals could anticipate rewards even when cues were not directly tied to them, showcasing a broader impact of Pavlovian conditioning and raising the idea of generalized ‘conditioned anticipation’. This first effort towards understanding how Pavlovian and instrumental learning converged was provided by Rescorla and Solomon's two-process theory [3], followed by more systematic works by LoLordo *et al*. [4], Schwartz [5] and Lovibond [6]. In a parallel line of research, Brown & Jenkins [7] suggested that Pavlovian conditioning can influence animals' instrumental behaviour even in the absence of any direct reinforcement.

Pavlovian influence refers to the fact that animals (non-human as well as human) show a predisposition to act (e.g. salivate, approach) when environmental cues (e.g. a sound) promise a positive outcome (i.e. food). Similarly, we tend to freeze or withdraw when the cues around us tell us that bad things are likely to follow. Though we are prone to approach when we see an opportunity and pass when we see a threat, there is, however, no *a priori* reason why acting should be more likely to be associated with positive outcomes and not acting (or avoiding) with negative ones. One can find examples of actions leading to negative outcomes (e.g. eating a bitter food) as well as examples where inaction or avoidance leads to positive outcomes (e.g. not moving and getting a treat for staying still). Nonetheless, extensive research in animal behavioural sciences shows that these associations are easier to learn especially compared with the association between not acting and reward [7].

One of the clearest laboratory models for Pavlovian influence in human decision-making is the orthogonalized Go–NoGo task that was introduced by Marc Guitart-Masip *et al.* [8]. Their design combined the features of classical Pavlovian conditioning—which associates a cue with a reward or punishment—with those of instrumental learning—which associates an action with a reward or punishment. Each trial started with an image, acting as a visual Pavlovian cue. Altogether, there were four associations to learn. Two cues promised a win: if the participant succeeded, she would make some money but win nothing if she failed. The other two cues promised a loss: if the participant failed, she would lose some money and lose nothing if she succeeded. After the cue, the participant was shown a circle target detection task and had to choose whether to perform a Go (active choice of pressing right or left keys to indicate the side of the circle) or a NoGo (passive choice of not pressing anything). This part corresponded to the instrumental component of learning. The overall performance depended on learning how to react to each cue, that is, to do something or do nothing. The presentation of cues was random. The participants had to figure out and keep track of what each cue was associated with and respond accordingly to maximize their monetary gain.

**Figure 1.**
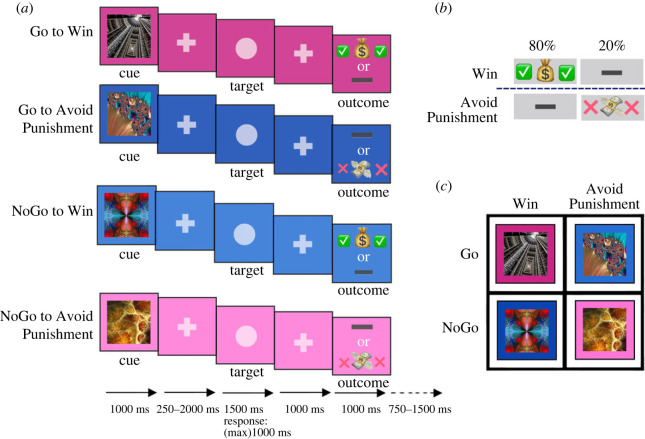
Experimental design. (*a*) Schematic illustration of the sequence of events in the four conditions. (*b*) Probabilistic outcomes for a correct decision. In the Win trials, if the participant decided correctly, there would be an 80% chance of a monetary reward and 20% chance of receiving nothing. In the Punishment trials, if the participant decided correctly, there would be an 80% chance of losing nothing and a 20% chance of losing some money. (*c*) An example assignment of four cue images to the 2 × 2 table of action (rows) and outcomes (columns).

Guitart-Masip and colleagues' findings showed that after several blocks of trial and error, participants were nearly perfect when the cue indicated that pressing a button earned them some money (Go-to-Win) [8]. They were not as good but still better than chance and nearly equal in two intermediate conditions: when pressing a button helped them avoid losing (Go-to-Avoid-Punishment) and when they should have withheld from pressing a button to avoid an upcoming punishment (NoGo-to-Avoid-Punishment). This latter result confirmed that people were able to learn when to do nothing to avoid a loss. Interestingly, people were worst, in fact on average nearly at the chance, in learning when they should refrain from pressing the button in order to earn money (NoGo-to-Win). Many participants in the experiment failed to learn that there was an occasion to succeed by avoiding an opportunity. The combination of high performance in the average of two conditions (Go-to-Win and NoGo-to-Avoid-Punishment) and low performance in the average of the other two shapes the Pavlovian bias. In other words, in this paradigm Pavlovian bias is quantified through the cross-over interaction in the 2 × 2 table depicted in figure 1*c*.

While many studies have replicated these results [8–14], they consistently show a large diversity among individual participants when it comes to the most challenging part, i.e. when doing nothing can lead to reward. In these replications, one can find nearly perfect participants, showing a perfect capacity to hold back from pressing a button when anticipating a reward. In the same studies, one can also find participants that cannot help but press some button in response to the reward cue. A full range of performances spread between these two ends, showing that people vary considerably in their ability to learn the association between reward cues and inaction.

Computational psychiatry aims to translate the advances in computational neuroscience into concrete improvements for patients suffering from mental illness. One particularly fruitful research method in computational psychiatry is to search for parametrizations of a given psychological concept that define individuals by their placement in some multidimensional parameter space. If healthy individuals and those manifesting a certain category of mental health disruption aggregate to distinct areas of such space, then we have found external validity for the computational articulation of that psychological concept. Importantly, a key prerequisite for the validity of the findings in this approach is to demonstrate test/retest reliability. The interindividual differences captured by the given computational method should be stable across time before we could seriously consider them. A number of previous works have applied this approach to Pavlovian influence on learning, hoping to identify the latent variables of decision-making that can be linked to mental health. These works have demonstrated the translational relevance of Pavlovian influence for clinical research on anxiety [15], post-traumatic stress disorder [16] and depression [17], offering encouraging evidence for the external validity of Pavlovian bias. However, establishing empirical evidence for the temporal stability of these individual differences has proven difficult [13].

The null hypothesis here is that the diversity in performance may be nothing other than a measurement error. After all, as going against Pavlovian influence is hard, behaviour measurements may produce a very noisy result in this condition. In such a case, the variability between people would not tell us anything about them. Repeating the same experiment with the same group of people should then produce two uncorrelated sets of results: a person who participated on one day and did very well may totally fail to do as well the following week.

By contrast, if individual learning differences are not merely due to measurement noise, and indicate stable differences between people, repeating the same experiment with the same group of people should produce correlated sets of results: how a person performed on one day should give a clue about how she would perform in a second session.

A previous work [13] examined the question of the temporal stability of Pavlovian bias in adolescents and young adults (age range: 14–24). The results showed weak evidence for the stability of Pavlovian influence across time. Going about this research question in a comprehensive manner, Moutoussis and colleagues tested two different groups of participants across two different time intervals: the first group was retested after six months, and the second after eighteen months. Puzzlingly, interindividual differences in the first group showed no evidence of stability or consistency. Data from the second group did show some, albeit weak evidence (correlation coefficient approx. 0.15). Note that the expectation of *r* > 0.70 is the typical lower cut-off used for assessing the reliability of self-reported measures in psychometrics. These results were inconclusive and more consistent with the measurement noise account of interindividual differences, casting doubt on the usefulness of the Go–NoGo paradigm in translational research.

Given the strong evidence for external validity of this paradigm [8,10–17], we revisited this question with a number of key changes to the experimental protocol and a pre-registered design (https://osf.io/rndpf). Our hypothesis was that these modifications would provide stronger evidence for temporal stability of interindividual differences in Pavlovian bias.

To briefly recap, this task consists of instrumental learning of active and passive choices (Go and NoGo) that are reinforced with probabilistic outcomes (win, lose or nothing), where the best choice (Go or NoGo) is indicated by the visual cue and independent of the outcome (win or lose). We pre-registered our study with a new, web-based set-up, an entirely adult participating population, and a shortened interval between Test and Retest sessions. The setting of our study was changed to an online experiment, recruiting participants through Amazon TurkPrime. The experiment's graphics were gamified and the instructions were carefully reviewed to enhance the communication with the participants. We restricted our participant group to adults aged over 18, in two sessions (called ‘Test’ and ‘Re-Test’). Finally, in order to make the most of the Test/Re-Test arrangement in terms of its compactness and credibility, we came to the conclusion, based on previous research [18–20], that a two-weeks window could be a reasonable interval between the Test and the Re-Test sessions.

## 2. Methods

### 2.1. Pre-registration

All pre-registration material can be found in the Open Science Framework project https://osf.io/rndpf.

### 2.2. Participants

We recruited adult US residents (age greater than or equal to 18) from Amazon Mechanical Turk (Test: *N* = 202, Re-Test: *N* = 130). The sample size was estimated by following previous, similar online studies [21–23]. After applying the exclusion criteria, 146 participants were selected for the Test and 114 for the Re-Test (see electronic supplementary material, table S1). Therefore, the final sample size to examine the test–retest reliability was *N* = 114 (table 1).

**Table 1.** Key descriptive characteristics of participants at Test and Re-Test sessions.

	total participants after exclusion	gender after exclusion		age after exclusion	
Test	*N* = 146			mean	39.4
		male	83	Q1	30
		female	59	Q3	46
		other	4	range	(22, 71)
Re-Test	*N* = 114			mean	39.24
		male	65	Q1	31
		female	45	Q3	46
		other	4	range	(22, 71)

### 2.3. Orthogonalized Go–NoGo task

To measure an individual's Pavlovian bias as the main variable of our study, we used a variant of the ‘Orthogonalized Go–NoGo task’ which was first described by Guitart-Masip *et al.* [8]. This task can dissociate the tendency to do something (Go) rather than do nothing (NoGo) for receiving a ‘reward’ or avoiding a ‘punishment’. The resulting 2 × 2 design (figure 1*c*) consists of four conditions: Go to Win (G2W), Go to Avoid Punishment (G2AP), NoGo to Win (NG2W) and NoGo to Avoid Punishment (NG2AP).

Each trial (figure 1*a*) started with a cue displayed for 1000 ms. After the cue, a fixation cross was presented in the middle of the screen for a random period between 250 and 2000 ms. Next, a ‘target’ circle was presented on the screen, placed randomly to the left or right of the centre, for 1500 ms. At this point, the participant had to decide between Go (indicate the circle's left/right position by button press) or NoGo (do nothing) for 1000 ms. Finally, the trial ended with the presentation of the outcome for 1000 ms. When the subject won, a dollar bag and two green check mark emojis (figure 1*b*) were displayed. When the subject lost, a flying dollar bill surrounded by two red cross mark emojis was presented. When the subject won or lost nothing, a rectangular emoji cue was displayed.

Thus, in each trial, participants saw one stimulus from a set of four different stimuli (figure 1*c*), each of which indicated one of the combinations between action ('Go’ or ‘NoGo') and outcome ('reward’ or ‘avoid punishment'). Based on this cue, the participant made a decision—which could be either to press (Go) or withhold (NoGo)—when the target circle appeared to the left or right of the screen. If participants took the correct decision, the better outcome (figure 1*b*) occurred with a fixed probability of 80%. Depending on the cue, the better outcome was either ‘reward’ (against ‘nothing’) or ‘nothing’ (against ‘punishment’).

To test the reliability of our online set-up, we conducted two pilot studies. In the first pilot, the original, longer version of the task was conducted for eight participants. Following that, we simplified the instructions and added some visual guides for carrying out the experiment in the second pilot. The modified version was then run for nine participants on Amazon TurkPrime. We observed that participants were losing focus during the task, and some complained that they did not understand the task. We modified the task by (i) shortening the duration of the experiment, (ii) adding a cautionary statement about focusing and not changing the tab/window during the task, and (iii) designing a quiz test about instructions (see electronic supplementary material, S1). As a result of these modifications, several slight adjustments were made to the final task compared with the previously published original versions of the task [8,13]. To see the details of the experiment, we encourage the reader to try the demo version of the task themselves at sepsad.github.io/Orthogonalized-goNoGo-Task.

In this final setting, there were 30 trials for each of the four conditions (a total of 120 trials) divided equally into three blocks, 40 per block (it was less than the original task, 240 trials, 60 trials per condition divided into four blocks). Four fractal images were used as cues. The correspondence between fractals and conditions was assigned randomly for each participant. Each cue appeared 10 times in each block in random order. Participants were informed about the outcome of each trial by emoji icons. We displayed ‘green checkmark’ and ‘money bag’ emojis 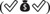 to represent winning and monetary rewarded outcomes, ‘red cross mark’ and ‘money with wings’ emojis 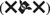 to represent losing and monetary punished outcomes, and a ‘heavy minus sign’ emoji 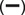 to represent neutral (no reward/no punishment) outcomes. After each block, participants took an up to 1 min break. Before starting the main part of the task, participants read the instructions, took a quiz to test their understanding of the instructions and practised 12 trials in order to get familiarized with the task. Participants received $5 to complete the task regardless of their performance, with a $2 additional bonus linked to their performance instructions (see electronic supplementary material, S1). The task was coded in jsPsych [24], version 6.3.1.

### 2.4. Procedure

The study consisted of two sessions, Test and Re-Test, which were separated by a two-week interval. In each session, we administered the orthogonalized Go–NoGo task. Participants in the Test who successfully passed the performance criteria were invited back for the Re-Test session via email. In the Re-Test session, we used a different set of fractals from the Test session. Exclusion criteria were (i) more than 45% error rate in target circle localization performance (figure 1*a*) (*N* = 44 were excluded) and (ii) pressing no keys or the Go response throughout the experiment in all trials (*N* = 12 were excluded). The detailed demographics of the participants are listed in electronic supplementary material, table S1. The procedure described here was part of a larger study (cf. the pre-registered document osf.io/rndpf), which included another third session for questionnaire one week after the Re-Test session. The current paper does not concern the third session.

One limitation of our study is that we did not conduct an *a priori* power analysis before data collection. The goal of our study was to evaluate the test–retest reliability of our measure of Pavlovian bias. This would mean that, in addition to rejecting the null hypothesis of *r* = 0, we were interested in accurately estimating the confidence interval around the test–retest correlation. Given the previously available data from Moutoussis *et al.* [13] in which *r* = 0.15, our sample size of *N* = 114 would afford a precision of [−0.035, 0.33]. Doing the same calculation for what the study's best hopes aimed to achieve, i.e. test–retest reliability greater than 0.7, our sample size would afford a confidence interval of [0.59, 0.78] giving us an estimate of the best-case scenario. Inevitably, our empirical data fall short of this hopeful ideal. Indeed, our paradigm did include new modifications (online experiment, two-weeks time window, adult population) that diverged from the previous works and limited the utility of such estimates. Since our report was submitted for review, two other preprints have also appeared online [25,26] offering their own datasets and corroborating our findings regarding the consistency of Pavlovian bias across time. We encourage the researchers who wish to use our paradigm to take into account all of these data to obtain *a priori* sample size estimates.

### 2.5. Computational models

To assess the behaviour of each participant, their data were modelled using a set of variants of the core reinforcement learning model (Rescorla–Wagner) employed in previous studies. The models are able to dissociate the underlying processes of learning in the task and allow us to test the reliability of components observed in choice behaviour, specifically Pavlovian biases on action-value computation throughout the experiment.

In all models, the values of each action (*Q* values) were calculated for all conditions, and a SoftMax choice function was used to assign an action probability on each trial. The core model included two free parameters for outcome sensitivity (rho) and learning rate (alpha). Depending on the condition, feedback (reinforcements) (*r*) would take the form of (1, 0, −1) that indicated reward, nothing (neutral outcome), and punishment, respectively. State-action values (*Q* values) were updated according to the simple Rescorla–Wagner rule

2.1 $$Q_{t}(a_{t},s_{t})=Q_{t-1}(a_{t},s_{t})+\alpha (\rho r_{t}-Q_{t-1}(a_{t},s_{t})).$$

State-action values were calculated differently in different models, and different parameters were included in those models sequentially. The first parameter added to the core model was irreducible noise in action selection in order that the possibility that some trials might not be selected was taken into account, as follows:

2.2 $$p(a_{t}|s_{t})=\left[\frac{\exp(W(a_{t}|s_{t}))}{\sum_{a^{\prime }}\exp(W(a^{\prime }|s_{t}))}\right](1-\xi )+\frac{\xi }{2}.$$

A second parameter was a bias to take action (Go) which described the tendency to press the button, as follows:

2.3 $$W_{t}(a,s)=\begin{array}{cc} Q_{t}(a,s)+b & \mathrm{if}\;a=\mathrm{go}\\ Q_{t}(a,s) & \mathrm{else}. \end{array}$$

A third and most crucial parameter was Pavlovian bias, which determined the degree to which action was invigorated for conditions associated with reward and suppressed for conditions associated with punishment. This term (*π*) was multiplied by State value *V* as a Pavlovian factor and then added to the action value (Q(Go)).

2.4 $$W_{t}(a,s)=\begin{array}{cc} Q_{t}(a,s)+b+\pi V_{t}(s) & \mathrm{if}\;a=\mathrm{go}\\ Q_{t}(a,s) & \mathrm{else}. \end{array}$$

The State value *V* of each condition was estimated in two ways; First, in each trial in a similar procedure to *Q* value estimation (pav)

2.5 $$V_{t}(s_{t})=V_{t-1}(s_{t})+\alpha (\rho r_{t}-V_{t-1}(s_{t})).$$

And second, in each trial, the sign of equation (2.5) (+ 1 or −1) is used for the current state value (pav(const))

2.6 $$V_{t}(s_{t})=\mathrm{sgn}\;(V_{t-1}(s_{t})+\alpha (\rho r_{t}-V_{t-1}(s_{t}))\;).$$

In the model (RW (rew/pun) + noise + bias + pav), the Pavlovian parameter inhibits the tendency to go in conditions where the outcome is punishments. Similarly, it encourages the tendency to go in situations where the outcome is positive. This inhibition/invigoration is proportional to magnitude of *V*(*s*) (equation (2.5)). In the other model with the Pavlovian influence, (RW (rew/pun) + noise + bias + pav(const)) the influence on inhibition/invigoration is constant and only depends on the sign but not the magnitude of *V*(*s*) (equation (2.6)).

Finally, A fourth parameter accounted for potentially different sensitivity to reward versus punishment (rho_rew, rho_pun) that captured an asymmetry in the impact of rewards and punishments.

### 2.6. Model fitting

As in previous, similar studies [8,10–14] using this model, the model fitting procedure and individuals' parameters estimation were conducted using the expectation-maximization method. For each participant, the maximum *a posteriori* estimation for the parameters is calculated iteratively using the Laplacian approximation. To control individuals’ parameters reaching extreme values, after an iteration, the mean posterior and variance of each parameter are used as prior distributions for parameter maximization in the next iteration. The algorithm finishes when it converges to the near-identical parameter value.

To compare models, integrated Bayesian information criterion (iBIC) was calculated as in the previous studies [8,10–14]. While the BIC estimates the penalized individual-level likelihood of the data given a set of parameters, the iBIC calculates the penalized group-level likelihoods over the estimated distribution of the group-level hyperparameters. Lower iBIC values indicate a model that fits better after penalizing the number of parameters. This method enabled us to compare models with different parameters.

Following previous studies [8,10–14], Pavlovian bias and outcome sensitivities were constrained to be between 0 and ∞, learning rate and irreducible noise were constrained to be between 0 and 1, and the action bias parameter was unconstrained. The models were fitted using MATLAB [27].

### 2.7. Data analyses

Statistical analyses were performed using Statsmodel 0.13.0 [28] and Scipy 1.6.0 [29] packages on Python 3.9. As mentioned before, the focus of the present study was on the test–retest reliability of Pavlovian. And subsequent to using the entire data from all four conditions and employing the model to assess behavioural parameters, we used a Pearson correlation to examine if Pavlovian bias and other parameters were correlated between the two sessions.

## 3. Results

### 3.1. Online replication

**Figure 2.**
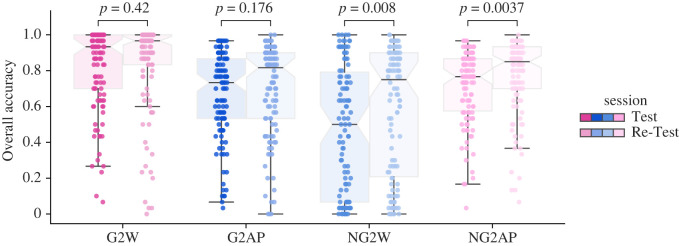
Raw performances comparison between Test and Re-Test session. Blue: Pavlovian incongruent (NG2W, G2AP) condition. Pink: Pavlovian congruent (G2W, NG2AP). The non-overlapping notches represent an uncorrected difference between two medians of *p* = 0.05. Left. Test (*N* = 114): The pattern of the overall accuracy in all conditions looks like the previous studies, and, in No Go to Win (NG2W) trials, the median performance is slightly lower than chance. Right. Re-Test (*N* = 114): Re-Test experiment generally showed better performance especially in NG2W condition. Still, in the NG2W condition at least a quarter of participants performed worse than chance (first quartile of Re-Test NG2W accuracy = 0.21).

In our setting, participants were tested using two online sessions. In each session, individuals participated in a web-based version of orthogonalized Go–NoGo task, and they were tested without any formal lab constraints. We analysed subjects' performances based on the proportion of correct responses in each task condition. A two-way ANOVA on Test's performance with action and valence factors revealed a main effect of action (*F*_1,145_ = 42.47, *p* = 0.001) and action by valence interaction (*F*_1,145_ = 69.80, *p* = 0.001) but no main effect of valence (*F*_1,145_ = 1.40, *p* = 0.237). The results of a similar two-way ANOVA on the Re-Test's data showed a main effect of action (*F*_1,113_ = 12.35, *p* < 0.001) and an action by valence interaction (*F*_1,113_ = 43.08, *p* < 0.001) but no main effect of valence (*F*_1,113_ = 0.67, *p* = 0.413). Figure 2 demonstrates that Pavlovian-incongruent conditions (NG2W and G2AP) had worse performance than congruent ones (G2W and NG2AP). Moreover, raw performances show that both rewards and punishments were effective in subjects' learning, but the ‘Go’ options outperformed the ‘NoGo’ options. These patterns are similar to previous studies [8,10–14], and the ‘Pavlovian bias’ interaction pattern was seen in both online experiments.

Next, we conducted a three-way ANOVA with factors of action (Go/NoGo), valence (reward/punishment) and sessions (Test/Re-Test). It showed a main effect of action (*F*_1,113_ = 45.140, *p* < 0.001), a main effect of sessions (*F*_1,113_ = 11.986, *p* < 0.001), and an action by valence interaction (*F*_1,113_ = 74.369, *p* < 0.001).

To compare performances in each condition between Test and Re-Test sessions, we conducted a pairwise *t*-test for each condition. Results showed significant improvements in the NG2W condition (figure 2: *t*_113_ = 2.71, *p* = 0.008) and the NG2AP condition (figure 2: *t*_113_ = 2.97, *p* = 0.0037) but not for the G2W (figure 2: *t*_113_ = 1.362, *p* = 0.176) nor G2AP condition (figure 2: *t*_113_ = 0.81, *p* = 0.42).

Also, we conducted an *F*-test for equality of variances between Test and Re-Test accuracy data in each condition. Results showed that in each of the four conditions, variance was not different when comparing Test and Re-Test sessions (figure 2: in NG2W conditions, *F*_113,113_ = 1.003 with *p* = 0.495; in G2W conditions, *F*_113,113_ = 0.84 with *p* = 0.81; in G2AP conditions, *F*_113,113_ = 0.86 with *p* = 0.78; and in NG2AP conditions, *F*_113,113_ = 0.95 with *p* = 0.61).

### 3.2. Stability of performance in each condition

**Figure 3.**
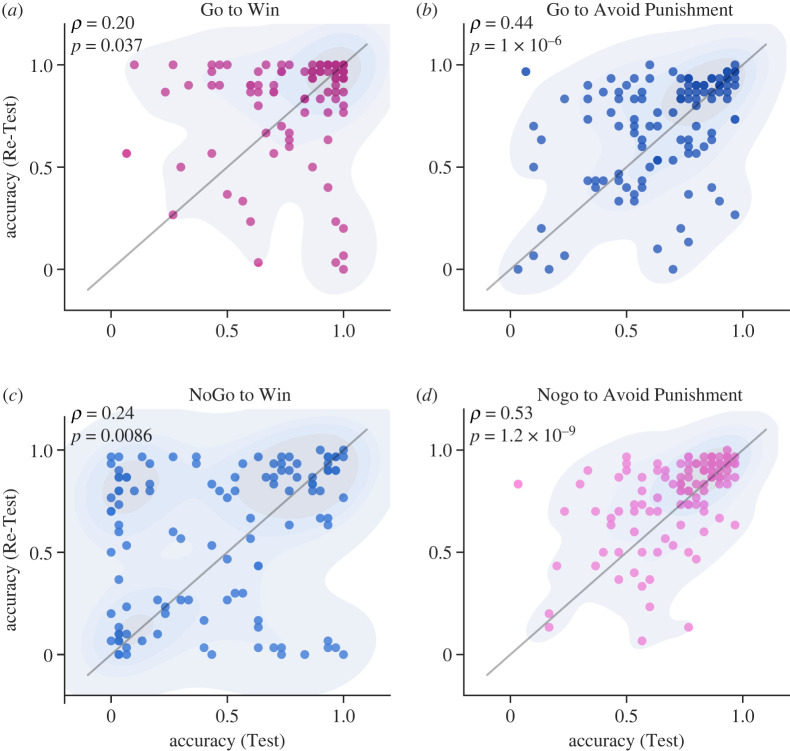
Within each condition performance accuracy in Test and Re-Test session were correlated. In each panel, the X and Y axes show accuracy in the Test and Re-Test sessions (*N* = 114), respectively. Pavlovian-congruent conditions (*a,d*) are illustrated in pink and Pavlovian-incongruent ones *(b,c*) in blue. In all panels, the diagonal line is the identity line, not a data-fitting line. Curved lines are kernel-density contours. A significant correlation is found in all four conditions. There is conspicuously more variability in panel (*c*), the NoGo to Win condition.

To examine the test–retest reliability of Pavlovian bias, we tested participants in the Test and then after intervals of two weeks in the Re-Test using the orthogonalized Go–NoGo task (see Methods). First, we investigated the stability of the performance of each condition and tested whether the performances were stable with task repetition. Figure 3 shows there was a significant correlation between performance accuracy at Test and Re-Test. Test session accuracies were found to have a significant correlation with Re-Test session accuracies in all four conditions. Specifically, in the NG2W conditions, *r*_112_ = 0.24, *p* = 0.0086, 95% CI (0.059, 0.41). In the NGAP conditions, *r*_112_ = 0.53, *p* < 0.001, 95% CI (0.38, 0.65). Similarly, in the G2AP conditions, *r*_112_ = 0.44, *p* < 0.001, 95% CI (0.28, 0.58) and in the G2W conditions, *r*_112_ = 0.20, *p* = 0.037, 95% CI (0.017, 0.367).

To further assess the stability of the interindividual differences over time and go beyond the raw accuracy measures, we calculated the descriptive estimates of Pavlovian bias and Go bias using a linear composition of the performance accuracy in the four conditions such that

3.1 descriptive estimate of Pavlovian bias=(G2W+ NG2AP)−(NG2W+G2AP),

3.2 descriptive estimate of Go bias=(G2W+G2AP)−(NG2AP+NG2W).

**Figure 4.**
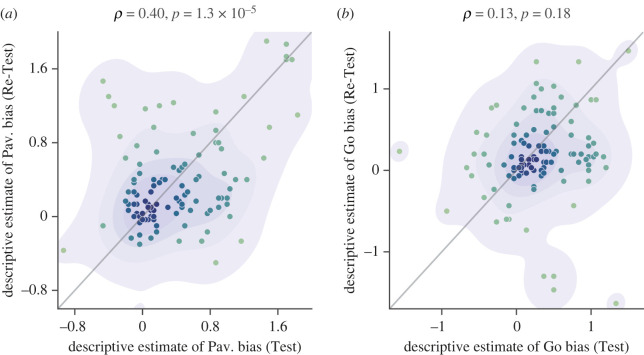
Stability of the descriptive estimates of (*a*) Pavlovian bias and (*b*) Go bias across the Test and the Re-Test sessions. Descriptive estimates of Pavlovian bias are calculated according to equation (3.1) and descriptive estimates of Go bias are calculated according to equation (3.2). Contours and colours show kernel density. Note that the diagonal is the identity line and not any fit to the data.

A significant Pearson correlation between the descriptive estimates of Pavlovian bias obtained from the Test and the Re-Test (figure 4*a*: *r*_112_ = 0.40; *p* < 0.001; 95% CI (0.23, 0.54)) indicated high consistency across time. Nonetheless, the Pearson correlation between the descriptive estimates of Go bias across the Test and Re-Test sessions was not statistically significant (figure 4*b*: *r*_112_ = 0.13; *p* = 0.18; 95% CI (−0.059, 0.30)).

### 3.3. Modelling results

**Figure 5.**
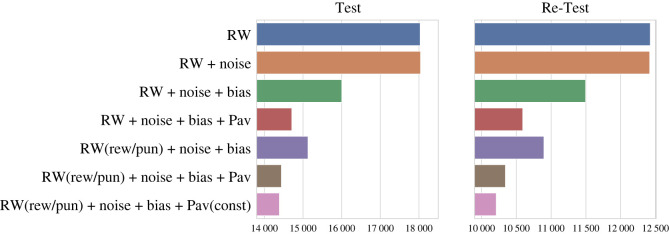
Model comparison for Test and Re-Test experimental data. Integrated Bayesian information criterion (iBIC) for all models fitted is plotted for the Test (left) and Re-Test (right) experiments. All models are extensions of the Rescorla–Wagner model with a pair of action values (i.e. Go and NoGo) for each state (i.e. cue image). The winning model (the bottom row) for both sessions includes different learning rate for reward and punishment, an irreducible noise, a constant action bias factor, and a Pavlovian factor that adds a fraction of the current state value (the reward of the previous trial) to the action value for Go.

Following previous works [8,10–14], to distinguish the roles of the underlying cognitive processes engaged during the experimental task, we fitted a set of variants of the Rescorla–Wagner model. To compare models, we used an iBIC. In both Test and Re-Test experiments, the (RW (rew/pun) + noise + bias + pav(const)) performed best, with 53.2 iBIC units and 131.7 iBIC units over the second-best model, respectively figure 5.

**Figure 6.**
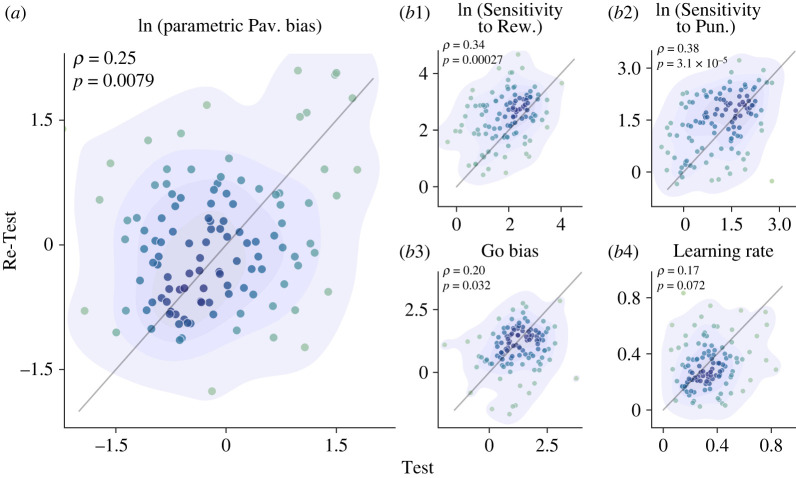
Model parameters' stability over time. In Each graph, X and Y axes refer to the model parameter estimated for the Test and the Re-Test (*N* = 114), respectively. The label above each graph indicates the relevant model parameter. (*a*) Logarithm of Pavlovian bias. (*b*1) Logarithm of sensitivity to reward. (*b*2) Logarithm of sensitivity to punishment. (*b*3) Go bias. (*b*4) Learning rate. Statistically, a significant correlation is seen for all mentioned parameters except for the learning rate *(p* = 0.07).

For parametric Pavlovian biases, the Test experiment's metric was significantly correlated with the Re-Test's metric (*r*_112_ = 0.25; *p* = 0.0079; 95% CI (0.069, 0.41): figure 6*a*). Correspondingly, it was true for other parameters of the winning model except for the learning rate. The sensitivity to reward and punishment, which measures how vigorously a choice can be influenced by reward and punishment, had (*r*_112_ = 0.34 with *p* < 0.001; 95% CI (0.17, 0.49)) and (*r*_112_ = 0.38 with *p* < 0.001; 95% CI (0.21, 0.53)), respectively. For the overall propensity to act, Go bias, had *r*_112_ = 0.20 with *p* = 0.032; 95% CI (0.017, 0.37). In the case of the learning rate, the correlation was marginally below significance level (*r*_112_ = 0.17, *p* = 0.072; 95% CI (−0.14, 0.34)).

**Figure 7.**
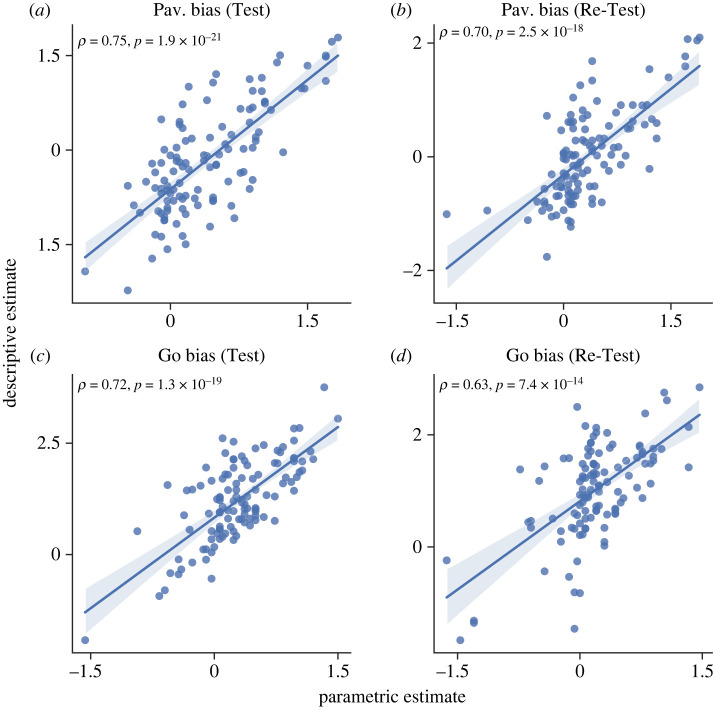
Assessment of the winning model quality. Parametric and descriptive estimates of Pavlovian and Go biases are consistent. In each graph, X axes refer to one parameter estimated by the model and Y axes refer to the corresponding descriptive estimate driven directly from the behaviour. The label above each graph indicates the relevant model parameter and session. (*a*) Pavlovian bias in Test session (*b*) Pavlovian bias in Re-Test session (*c*) Go bias in Test session (*d*) Go bias in Re-Test session.

To assess modelling quality and for a sanity check, we conducted a Pearson correlation test between parameters extracted from the winning model and the corresponding descriptive estimates calculated earlier (equations (3.1) and (3.2)) within every session. The parametric measure of Pavlovian bias was found to be strongly correlated with the descriptive estimate of Pavlovian bias (in the Test session *r*_112_ = 0.75; *p* < 0.001; 95% CI (0.66, 0.82); figure 7*a*; in the Re-Test session *r*_112_ = 0.70; *p* < 0.001; 95% CI (0.59, 0.78); figure 7*b*). Similar findings were obtained for Go bias (in the Test session *r*_112_ = 0.72; *p* < 0.001; 95% CI (0.62, 0.80, figure 7*c*; in the Re-Test session *r*_112_ = 0.63; *p* < 0.001; 95% CI (0.50, 0.73), figure 7*d*).

## 4. Discussion

There is strong evidence for the external validity [12,15–17] of the Go–NoGo paradigm for measuring Pavlovian influence on learning. However, recent results from studies that examined this paradigm's temporal stability have been inclusive. This has cast doubts about the potential usefulness of this paradigm in computational psychiatry. We revisited the question of temporal stability of the Go–NoGo paradigm [13] with a number of key changes to the experimental protocol and a pre-registered design (https://osf.io/rndpf). Below, we summarize our key findings.

We replicated the classical findings of the Go–NoGo paradigm [8,10–14] in a web-based design that recruited online participants. We introduced a number of modifications to the instructions and some gamification to the set-up. Our online paradigm can now be used at much bigger scale than previous laboratory-based versions and this allows future studies to substantially increase their sample size, for example in clinical or genetic studies. We replicated the asymmetry for instrumental learning where individuals were better at learning to do something when they thought they would get a reward and better at learning to do nothing when they thought they would get punished. Consistent with the previous literature [7,8,12], doing nothing to get a reward was also the condition with the lowest average performance and highest interindividual variability among participants. This asymmetric pattern was preserved in the Re-Test data. Moreover, across the time window of two weeks, participants' performance improved in all four conditions of the experiment.

To address our main question, we employed two different methods to calculate the Pavlovian bias and found consistent results. We first calculated descriptive estimates of Pavlovian and Go biases (equations (3.1) and (3.2)) for each participant using the raw behavioural data (i.e. performance accuracy) from the Test and Re-Test experiments. Each of these estimates showed a significant temporal stability over the two sessions of the study. Next, following from previous works [1,10] we employed reinforcement learning (RL) to interpret the data. Five different models of progressively increasing complexity were constructed, fitted to individual participants’ data and compared through standard model comparison procedures. Our modelling results replicated the previous findings, converging to the same winning model as Moutoussis *et al.* [13] and Guitart-Masip *et al.* [8]. Focusing on the winning model, we then extracted the model-based estimate of the Pavlovian and Go biases for each participant. The model-based and descriptive estimates of Pavlovian and Go bias showed very strong consistency thus lending further support to the validity of our modelling exercise. Most importantly, compared with the previously reported results, the descriptive and model-based estimates of Pavlovian bias reported here showed considerably higher temporal stability (correlation coefficient *r =* 0.40 for descriptive and *r =* 0.25 for model-based) between the Test and Re-Test sessions. However, as noted in the introduction, the typical lower cut-off used for assessing the reliability of self-report measures in psychometrics is much higher (*r* > 0.70). It is therefore important to retain some caution when interpreting our results and treat them as moderate evidence for Pavlovian influence as a trait.

We chose a two-week time interval for the period between our Test and Re-Test sessions. This choice was informed by the fact that a previous study [10] employing longer intervals had not shown inconsistent results demonstrating no correlation with six-months period and some (albeit weak) correlation after 18 months. These paradoxical results, we reasoned, called for establishing a measure of consistency at some shorter interval as a baseline first before stretching the experimental paradigm to examine longer periods. Following from a number of previous works in the literature on individual differences in clinical psychology [17–19], we reckoned that two weeks would be a long enough period to serve as a reasonable minimum for recognition of stable individual differences. Given our positive findings, and the facility that online testing provides for large-scale individual difference studies, our results and the experimental paradigm now pave the way for future research to cover longer time intervals which would allow the direct comparison with previous works [10].

It is important to ask what factor may have contributed to our positive findings when another study [13] with a larger sample size failed to find evidence for temporal stability of Pavlovian bias. That study examined two, much longer time periods (six months and 18 months compared with our two weeks). The shorter time window is perhaps the most important reason why we have obtained our positive results. In addition to the time interval, there are a couple of other differences between the two studies. Moutoussis and colleagues tested a much more diverse range of participant age, spanning adolescent and adult populations. Their participants were, on average, 20 years old compared with ours who were, on average 39 years old. A number of previous studies have examined and some have found differences in learning ability in adolescent and adult groups [30]. Palminteri and colleagues compared reinforcement learning in adult and adolescent populations and found that adults employed more sophisticated learning strategies that involved counterfactual reasoning and value contextualization. Thus, it is possible that our study's focus on the adult population avoided a source of strategic variability and collected a more homogeneous dataset of behaviour giving rise to clearer findings. Finally, our experiment was gamified as an online experiment. In our pilots, we went through the experiment instructions step by step with a small number of participants and discussed with them what they thought the instructions meant and whether that coincided with what the experimenter had in mind. As a consequence, we made some editorial modifications to the way the instructions were presented and to the wording of the sentences. This meant that our experimental participants were somewhat differently instructed compared with standard practice of combination of verbal and printed instructions that is often given by the research assistants in laboratory experiments. We believe that these editorial modifications played a positive role in increasing the quality of our data without compromising the hypotheses. Our method, instructions and a demo of our experiment are all made available for interested research groups.

## Data Availability

The data and code that support the findings of this study are available at: https://osf.io/fnzd2/, https://github.com/Sepsad/Orthogonalized-goNoGo-Task. The data are provided in electronic supplementary material [31].
